# Caveats and Nuances of Model-Based and Model-Free Representational Connectivity Analysis

**DOI:** 10.3389/fnins.2022.755988

**Published:** 2022-03-10

**Authors:** Hamid Karimi-Rouzbahani, Alexandra Woolgar, Richard Henson, Hamed Nili

**Affiliations:** ^1^MRC Cognition and Brain Sciences Unit, University of Cambridge, Cambridge, United Kingdom; ^2^Department of Computing, Macquarie University, Sydney, NSW, Australia; ^3^Department of Psychiatry, University of Cambridge, Cambridge, United Kingdom; ^4^Department of Excellence for Neural Information Processing, Center for Molecular Neurobiology (ZMNH), University Medical Center Hamburg-Eppendorf (UKE), Hamburg, Germany; ^5^Wellcome Centre for Integrative Neuroimaging, University of Oxford, Oxford, United Kingdom

**Keywords:** representational connectivity analysis, multi-dimensional connectivity, functional connectivity, multivariate pattern analysis, representational similarity analysis

## Abstract

Brain connectivity analyses have conventionally relied on statistical relationship between one-dimensional summaries of activation in different brain areas. However, summarizing activation patterns within each area to a single dimension ignores the potential statistical dependencies between their multi-dimensional activity patterns. Representational Connectivity Analyses (RCA) is a method that quantifies the relationship between multi-dimensional patterns of activity without reducing the dimensionality of the data. We consider two variants of RCA. In model-free RCA, the goal is to quantify the shared information for two brain regions. In model-based RCA, one tests whether two regions have shared information about a specific aspect of the stimuli/task, as defined by a model. However, this is a new approach and the potential caveats of model-free and model-based RCA are still understudied. We first explain how model-based RCA detects connectivity through the lens of models, and then present three scenarios where model-based and model-free RCA give discrepant results. These conflicting results complicate the interpretation of functional connectivity. We highlight the challenges in three scenarios: complex intermediate models, common patterns across regions, and transformation of representational structure across brain regions. The article is accompanied by scripts (https://osf.io/3nxfa/) that reproduce the results. In each case, we suggest potential ways to mitigate the difficulties caused by inconsistent results. The results of this study shed light on some understudied aspects of RCA, and allow researchers to use the method more effectively.

## Introduction

To study the neural underpinnings of cognitive processes, we need not only to characterize the response of individual brain regions but understand the functional connectivity between them. This is critical to understand how brain regions interact in giving rise to cognition (Bressler and Menon, 2010). Functional connectivity across the brain has been conventionally evaluated using univariate/one-dimensional analyses (Bastos and Schoffelen, 2016). In these analyses, responses in each brain region is initially summarized by a one-dimensional metric (Biswal et al., 1995). If the 1D metrics for different regions are statistically related, we then infer functional connectivity between them (Bastos and Schoffelen, 2016). For example, methods such as gamma-band synchronization (Gregoriou et al., 2009), phase covariance across regions (Bar et al., 2006), frequency coupling (Karimi-Rouzbahani et al., 2021b), and differential equations (Friston et al., 2013) have been used to evaluate connectivity after summarizing the activation patterns across vertices (sensors or voxels) within each region. However, univariate connectivity analysis can miss connectivity if the pairs of regions are statistically related through multi-dimensional patterns of activation rather than the summarized (e.g., averaged) activation within each region (Coutanche, 2013; Basti et al., 2019, 2020). For example, for heterogeneous ROIs, where multiple response modes co-exist, projecting multivariate response patterns on a line (one dimension) could lead to strong distortions. This has led to a recent shift from univariate to multi-dimensional (multivariate) connectivity analyses (Coutanche and Thompson-Schill, 2013; Goddard et al., 2016; Anzellotti and Coutanche, 2018; Basti et al., 2019, 2020; Karimi-Rouzbahani et al., 2021a,c; Shahbazi et al., 2021). One approach to multi-dimensional connectivity is Representational Connectivity Analysis (RCA; Kriegeskorte et al., 2008), which utilizes the versatility of Representational Similarity Analysis (RSA) to move from the direct comparison of representations to the comparison of representational geometries (Kriegeskorte et al., 2008). Recent implementations of RCA can be divided into model-free [e.g., Information Flow Analysis (Goddard et al., 2016), RSA-Granger Analysis (Kietzmann et al., 2019), static RSA (Karimi-Rouzbahani et al., 2021c), and jackknife-resampling RCA (Coutanche et al., 2020)] and model-based (Clarke et al., 2018; Karimi-Rouzbahani et al., 2021a) methods, each having specific characteristics. Here, we describe model-free and model-based RCA and point out their differences. Specifically, we present three simple scenarios where model-free and model-based RCA provide inconsistent connectivity results, flagging the situations where they should be used with caution and adding nuance to how the results of each should be interpreted.

One key feature of RCA is that, rather than activations (Anzellotti et al., 2017a,b; Basti et al., 2019), it evaluates the statistical dependency between the geometry/structure of neural representations across areas. Accordingly, RCA relies on the distinctiveness (i.e., dissimilarity) of patterns across conditions, which is conceived in terms of “information encoding/representation,” rather than the activity patterns themselves. Therefore, one prerequisite for performing RCA is to have enough distinct experimental conditions to obtain the geometry of representations in the neural data [see, however, how we performed RCA on a single condition across time (Karimi-Rouzbahani et al., 2021c)]. This usually precludes RCA from being used to test functional connectivity in resting-state data (single, continuous fMRI, or M/EEG time series), which dominates univariate functional connectivity analyses. On the flip side, however, the representational nature of RCA provides several advantages over activity-based connectivity analyses. First, RCA allows the evaluation of connectivity across any two regions with different number of response channels (i.e., vertices, voxels, sensors, or sources; Kriegeskorte et al., 2008). Second, it allows one to ask how information (e.g., sensory, cognitive, etc.), rather than activation, is potentially transferred across areas. Third, model-based RCA allows one to target specific aspects of information, based on hypotheses about how a specific aspect of information is transferred, avoiding the influence from undesired confounders on connectivity (Clarke et al., 2018; Karimi-Rouzbahani et al., 2021a). Despite these advantages, under some circumstances representational connectivity analysis can miss true connectivity or erroneously detect non-existing (false) connectivity. This necessitates further investigation of RCA methods before they are more widely used as measures of multi-dimensional connectivity.

From a broad perspective, model-free RCA (Basti et al., 2020; Coutanche et al., 2020; Karimi-Rouzbahani et al., 2021c; Shahbazi et al., 2021) evaluates whether there is any commonality in the distributed patterns of activity for two brain regions. The commonality might reflect shared information due to similar encoding in the two regions. But it might also be due to the encoding of nuisance factors that are shared across regions. On the contrary, model-based RCA asks whether the two regions have shared information with regards to a specific hypothesis defined by a model. Having the model(s) can give a more specific picture of the multi-dimensional regional interactions. In this article, we compare model-free and model-based RCA and explain their pros and cons. In particular, we raise some cautions for using each method by showing simulated cases where one method fails to capture functional connectivity between two regions with shared information.

## Methods and Results

### General Simulation Details

**FIGURE 1:**
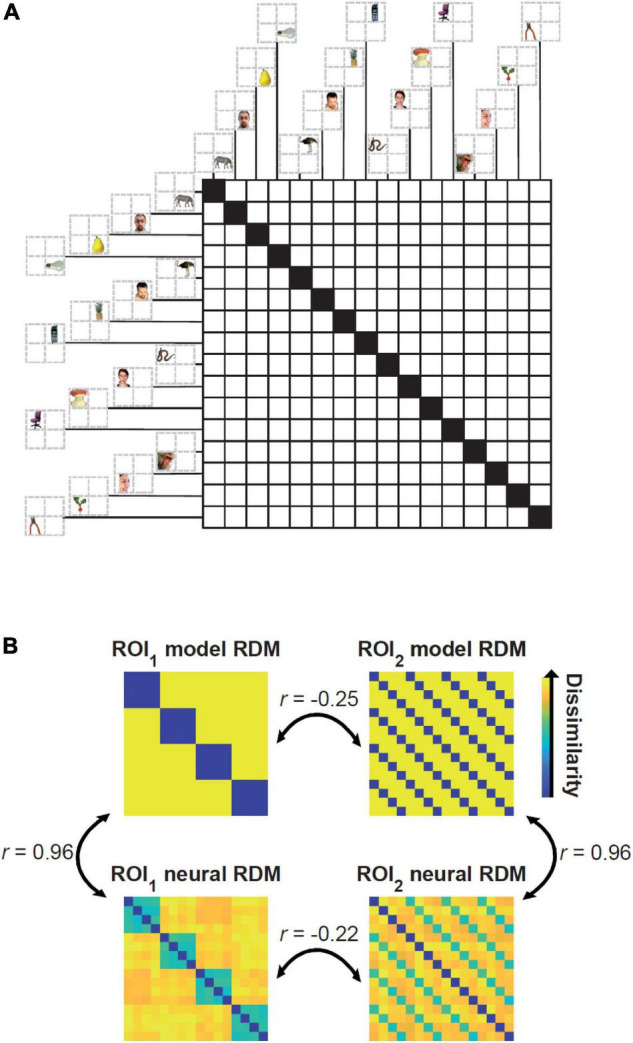
Arrangement of the simulated conditions in the representational dissimilarity matrices (RDMs), model and neural RDMs and their pairwise correlations. **(A)** There are 16 conditions in the RDM consisting of four semantic categories of objects in four distinct positions. Example stimuli are from Kriegeskorte et al. (2008) available at http://www.cns.nyu.edu/kianilab/ Datasets.html. **(B)** Top matrices show the ground truth for the two ROIs that represent “positions” (left) and “semantic categories” (right). The neural RDMs shown in the bottom were generated by adding noise to the activity patterns used to generate the two top model RDMs. Therefore, the simulated neural RDMs are highly correlated to the ground truth (*r* = 0.96). The correlations between the (model and/or neural) RDMs of the two different ROIs are negative (*r* < –0.2) implying no connectivity between them.

We generated multidimensional patterns of activity using scripts from the Matlab RSA toolbox (Nili et al., 2014; mean = 0; variance = 0.5; same statistics for every simulated subject). The Matlab script for reproducing the results can be downloaded from https://osf.io/3nxfa/. We simulated activity patterns for 16 stimuli in two brain regions. The number of vertices/voxels were set to 120 and 150 for regions of interest (ROIs) 1 and 2, respectively. The 16 conditions can be thought of as corresponding to four peripheral positions of the visual field (e.g., top left, top right, bottom left, and bottom right) of four semantically distinct visually presented object categories (e.g., animals, faces, fruits, and objects). For simpler explanation and interpretation of the results one can think of region of interest (ROI) 1 as visual area 2 (V2) and ROI 2 as inferior temporal cortex (ITC). Accordingly, ROI 1 dominantly represents position (i.e., regardless of the category of the objects) and ROI 2 dominantly represents semantic categories (i.e., regardless of the position of the stimuli). Figure 1A depicts the arrangements of the conditions in the Representational Dissimilarity Matrix (RDM). RDMs are generated by calculating the dissimilarity (here 1-correlation coefficient) of activity patterns across all experimental conditions and characterize the geometry of representations in the representational space (Kriegeskorte and Kievit, 2013). Figure 1B shows the ground-truth of the RDMs in the two ROIs and neural RDMs for a simulated subject which are different from the ground truths due to the added noise.

We used Pearson’s (linear) correlation for comparing RDMs. Accordingly, we only considered significantly positive correlations as indicating representational connectivity. We performed significance testing using a one-sided Wilcoxon’s signed rank test (Wilcoxon, 1992) across subjects and applied a threshold of 0.001 for statistical significance. Note that as RDMs are symmetric matrices, we only analyzed the elements in the upper triangle excluding the diagonal. We simulated data for *N* = 20 subjects to match it to the conventional number of subjects in real-life neuroimaging experiments and performed the statistical tests at group level.

### Simulation 1: Model-Based Representational Connectivity Analysis Tests Connectivity Through the Lens of Model(s)

#### Problem Statement

Model-based RCA is designed to test whether two ROIs are related with regards to a specific model^1^. A model privileges a specific direction in the dissimilarity space so that all comparisons would be made with respect to the direction specified by the model. This allows us to test whether two regions share particular information. Although there have been (two) different implementations of model-based RCA (Clarke et al., 2018; Karimi-Rouzbahani et al., 2021a), here we use a minimalistic implementation to raise concerns about caveats of model-based RCA as clearly as possible. A model-free approach to test for representational connectivity would be to directly compare the RDMs in the two ROIs. Here, we use linear correlation to perform model-free RCA.

**FIGURE 2:**
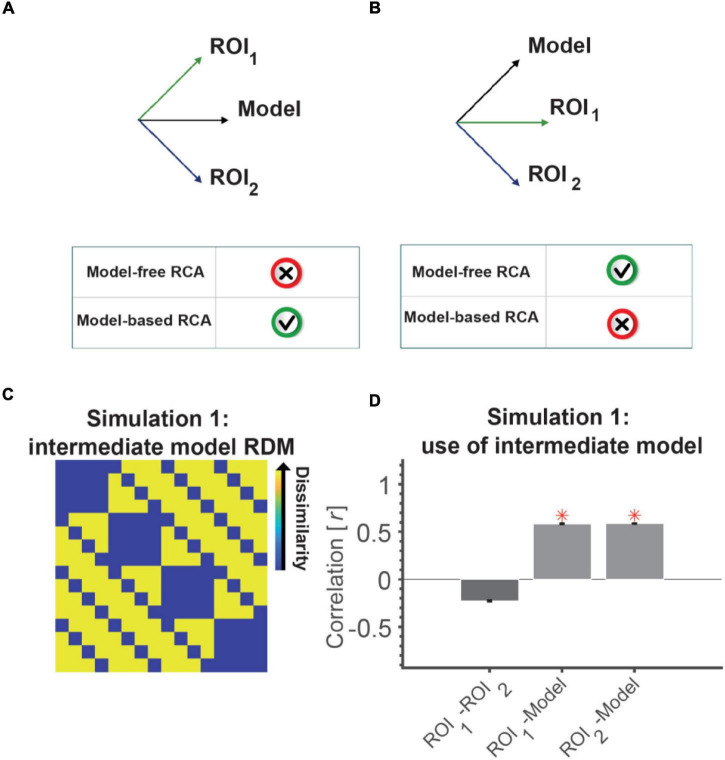
An intermediate model RDM can lead to the wrong conclusion that two uncorrelated ROIs (RDMs) are connected. RDMs can be represented as a vector emanating from the origin in the N-dimensional space [N = number of elements in the RDM, i.e., n(n–1)/2 with n being the number of conditions]. **(A)** Shows a situation where a model RDM has equal angles to two orthogonal (unrelated) neural RDMs. Looking from the lens of this model can leave the impression that the two neural RDMs are similar as they project similarly on the model RDM. **(B)** Shows a situation where two neural RDMs are positively correlated, but might look unrelated (disconnected) when looking at them through the lens of the model RDM. This is because only one has a component along the model RDM’s vector and the other is orthogonal. **(C)** The model RDM used in Simulation 1, which has components of the representations in ROI 1 (position) and ROI 2 (semantic category). This model RDM has equal correlations (= 0.6) to the models used to generate neural RDMs of the two ROIs (shown in Figure 1, top); almost similar correlations to the neural RDMs (0.6). **(D)** The correlation between the neural RDM of the two ROIs (model-free RCA) and between the neural and model RDMs of each ROI (model-based RCA). Red asterisks show significant above-chance correlation values (connectivity) as evaluated by a one-sided Wilcoxon’s signed rank test against zero.

Despite the potential benefits of model-based RCA, it has some limitations that should be considered with caution. For example, consider the scenarios depicted in Figure 2A (note that although RDMs can generally reside in a high dimensional dissimilarity space, we illustrate the main point with 2D figures). Figure 2A shows a case where RDMs from two ROIs have a positive correlation to a model RDM and in fact identical similarities to it (e.g., Pearson correlation of 0.7). Conceptually, model-based RCA asks whether correlations of two brain RDMs to a model RDM are similar. It would be tempting to conclude that two ROIs share the information captured by a model if they are equally close (similar) to it. However, in this example, the RDMs in the two ROIs are in fact orthogonal, so this conclusion would be erroneous. Unlike model-based RCA, model-free RCA (e.g., the correlation of the two RDMs) would correctly conclude no functional connectivity.

Conversely, consider the case depicted in Figure 2B. The neural RDMs in the two ROIs have a positive correlation (e.g., a Pearson correlation of 0.7). This means that model-free RCA would indicate representational connectivity. However, one ROI has no relationship to the model (correlation of 0) and another ROI has a positive correlation to it (*r* = 0.7), and therefore from the perspective of the model, the two ROIs would not be connected. While the two regions share some information, this is not the same information that is captured by the model.

These examples show that model-based and model-free RCA can easy lead to different results. It follows from the fact that model-free RCA is based on direct comparison of neural RDMs in the original dissimilarity space and that model-based RCA is based on comparison of projected RDMs on a line (projection), defined by the model. The degree of inconsistency depends on the neural RDMs and the direction defined by the model.

In Simulation 1 we present a scenario (similar to Figure 2A) where applying model-based RCA to two ROIs which represent independent aspects of visual stimuli could result in the wrong conclusion that the two are functionally connected.

#### Simulation Details

The general simulation details are provided in section “General Simulation Details.” For model-based RCA, we used a model RDM that incorporates both aspects of the stimuli (i.e., position and object category). This “intermediate” model hypothesized a larger pattern dissimilarity for two conditions that are different in both position and object category. The model had equal level of correlation/similarity to the neural RDM in each of the two ROIs (*r* = 0.6 between the model in Figure 2C and the RDMs shown in top and/or bottom panels of Figure 1). To implement model-based RCA, for each ROI we calculated the correlation between the neural RDM and the model for each subject. Then, we statistically compared the correlation results across subjects for the two ROIs. Specifically, two ROIs are considered to reflect model-based representational connectivity if they show significant positive correlations to a model and their correlations to the model are not significantly different. To implement the model-free RCA, we calculated the correlation between the neural RDMs of the two ROIs directly. Therefore, statistically positive correlation between neural RDMs shows model-free representational connectivity.

#### Simulation Results

Two ROIs that represent statistically unrelated information are not connected and do not have any shared information. However, looking at the two ROIs through the lens of an intermediate model in model-based RCA can leave the impression that the ROIs are connected. There was significant positive correlation for the two ROIs with the intermediate model, and the correlations between the model and the two ROIs were not statistically different (Wilcoxon’s signed rank test; *p* = 0.94, Figure 2D). This (incorrectly) suggests that the two ROIs are connected, by virtue of sharing the information captured in the intermediate model. However, model-free RCA (i.e., direct correlation between the two ROIs) correctly showed no positive correlation between the ROIs suggesting no connectivity. It might be worth adding that had we used a simple model instead of the intermediate model, for example, one of the two models illustrated in the top panel of Figure 1B, we would have correctly observed no model-based RCA. Therefore, the issue relates to the representational structures of the two ROIs as well as the model used for examining their connectivity.

#### Potential Solutions

To avoid false conclusion about connectivity across ROIs, it is important to evaluate it using both model-based and model-free RCA, see that if the results agree, and interpret accordingly. Where possible, for model-based RCA, it may also help to use minimal models where only one, rather than several, aspects of information is captured. In our simulation, the fact that our intermediate model had components from both aspects of stimuli (i.e., position and category) made it possible to capture variances explained by different processes, i.e., independent encoding of each aspect. Simpler models, for example models that correspond to simple hypotheses, might help to untangle representational connectivity along different dimensions of information transfer. However, it might be difficult to know these models in advance, unless the tasks are simple, and the underlying representations are already well characterized.

It is of note that, while we implemented a simplified version of RCA here, implementations in the literature have incorporated other parameters, such as time and delay, and other techniques such as multi-linear regression and partial correlation (Goddard et al., 2016, 2021; Karimi-Rouzbahani, 2018; Karimi-Rouzbahani et al., 2019, 2021a) each of which may affect the results. However, both the previous published implementations of model-based RCA (Clarke et al., 2018; Karimi-Rouzbahani et al., 2021a) ultimately rely on assessing the similarity in model fits between regions, so are subject to the concern we have demonstrated.

### Simulation 2: Spurious Connectivity From Common Input to Regions With Distinct Representations Can Be Avoided Using Model-Based Representational Connectivity Analysis With Appropriate Models

#### Problem Statement

There can be situations where common uninformative patterns are present along with the informative representations in the pair of ROIs considered for connectivity analysis. The common patterns can be as simple as measurement or neural noise which might be statistically dependent across areas and/or the leakage or feeding of activations from a third ROI to both ROIs as a result of proximity and/or poor spatial resolution (e.g., in EEG and MEG). On the other hand, it can also be the case that the two ROIs encode/represent some shared aspects, which are either task-irrelevant or not the target of study. For example, both position-selective early visual area (V2) and the semantically selective area (ITC) can be sensitive to low-level image statistics such as the spatial frequencies of the stimulus due to connections from V1. This shared information may lead to apparent connectivity if their RDMs are directly compared (as in model-free RCA), but may not reflect shared information of interest to the researcher. In general, we are not interested in capturing commonality in noise, and may not be interested in capturing this low-level information (i.e., spatial frequency) which are represented in both ROIs, but rather by the particular information for which we have hypotheses. In this simulation we ask whether model-based RCA is robust to this type of shared information and allows us to draw a specific conclusion about the shared information of interest to the researcher.

Below we simulate the impact of adding common patterns of activation to a pair of ROIs which otherwise represent distinct information, and show how model-free RCA, and some implementations of model-based RCA, can be affected. We show that using appropriate models that match the dominant representations of the two ROIs can mitigate the false connectivity.

#### Simulation Details

The neural patterns generated here are the same as Simulation 1 (with no connectivity between the two ROIs) except that now we also include the time course of representations to be able to implement more realistic model-free and model-based RCAs (rather than the simplified ones implemented in Simulation 1). We added the temporal dimension so that correlations could be computed over time. Please note that, however, ROI representations at different time points were consistent with the same structures depicted in Figure 1B. In other words, the information did not change over time but experienced some additive Gaussian noise (zero-mean; variance = 0.5). We simulated the activity patterns of the two ROIs over 200 time samples. The two ROIs were simulated to encode the two above-mentioned distinct aspects of information (i.e., position and semantic categories).

We performed model-free RCA by calculating the direct correlation between RDMs of the two ROIs at every time point and then averaging the resultant correlations over the simulated time window.

In this Simulation (and also the next simulation), we consider two versions of model-based RCA that have different motivations. In either case, we first obtained the correlation between the neural and the corresponding model RDM of each ROI at every time point and then calculated the correlation between the time courses of neural-model correlations for the two ROIs.

In the first version, we considered a common model for the two ROIs (similar to Simulation 1) and in the second version we used ROI-specific models (i.e., one model per ROI). The motivation for the first approach (1-model RCA) was that the experimenter might simply want to evaluate the information exchange reflecting a single known aspect of information (e.g., familiarity information across occipital vs. frontal areas: Karimi-Rouzbahani et al., 2021a). On the other hand, the experimenter might hypothesize that the dominant aspect of information represented in each of the two ROIs is different (e.g., visual information in lower visual areas vs. semantic information in ITC: Clarke et al., 2018). In this case it might be more suitable to compare each ROI to a specific model of itself (ROI-specific models) and use a 2-model RCA. In this case the interpretation of 2-model RCA results would be different and will be explained below (Simulation 3). For our implementation of 1-model RCA in the simulated example, we used the position model for both ROIs. For the 2-model RCA, we used the position model for ROI 1 and a semantic-category model for ROI 2; therefore, the models perfectly matched what was dominantly represented in each ROI.

**FIGURE 3:**
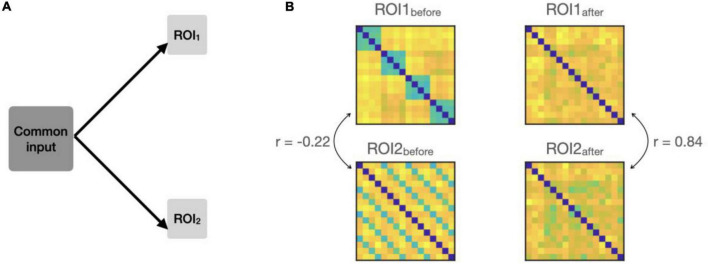
**(A)** Simulation settings, a common input is added to the responses in both ROIs, so that the patterns in the two ROIs covary at each time-point. **(B)** Example RDMs from the two ROIs before and after adding the common input. RDMs originally had a negative correlation (–0.22). The correlations went up from –0.22 to 0.84 due to the common input.

We added a non-structured (noise) pattern to both ROIs and evaluated its impact on connectivity (Figure 3A). To generate the common pattern, we used Gaussian noise (zero-mean; variance = 7) and a random transformation matrix (containing random numbers from a zero-mean unit-variance Gaussian distribution) to impose correlated noise across areas. Similar to Basti et al. (2020), we first simulated the added noise for one ROI and then transformed it via a multivariate linear mixing matrix to obtain the noise in the other ROI (Figure 3B).

#### Simulation Results

**FIGURE 4:**
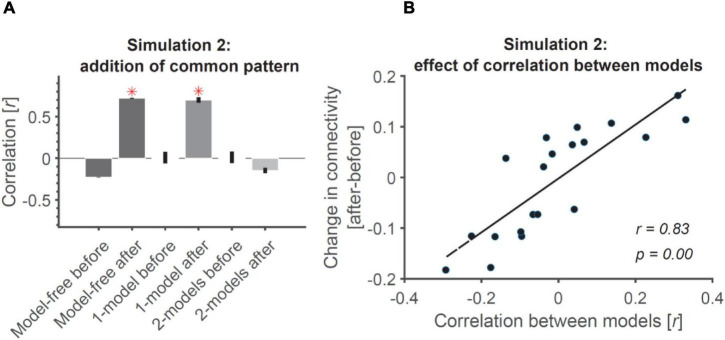
Addition of common pattern to pair of ROIs can make them look connected in model-free and 1-model RCA but not 2-model RCA if the ROIs are distinct enough. **(A)** Addition of common non-structured (noise) patterns to both ROIs leads to significant connectivity when evaluated using model-free and 1-model RCA, but not 2-model RCA because the two ROIs dominantly represent information that is negatively correlated (see Figure 1B). Red asterisks show significant above-chance correlation values (connectivity) as evaluated by one-sided Wilcoxon’s signed rank test against zero. **(B)** The more distinct the dominant information represented across the ROIs, the less the effect of added common noise on their connectivity. Dots show the amount of change in connectivity as a function of correlation between the information represented in the two model RDMs used in 2-model RCA. Each dot represents data from a single simulated subject. The line shows the best linear fit to the data. The correlation and the significance of correlations are also shown as calculated using Pearson’s linear correlation.

The results are shown in Figure 4A. As expected, before adding the common patterns to the ROIs, the three connectivity measures were either negative (model-free RCA) or around-zero (1-model and 2-models RCA), suggesting no connectivity between the ROIs (Figure 4A). However, the addition of common noise patterns to the two ROIs led to spurious connectivity for model-free and 1-model RCA, with both showing significantly above-chance connectivity. This was expected for the model-free RCA because it relies on shared information across ROIs, which become correlated by the added correlated noise (similar to the example depicted in Figure 3B). The researcher would conclude that the ROIs were connected, when in fact the positive result reflected shared information of no interest.

This result was especially interesting for 1-model RCA because the method required the two ROIs to be temporally correlated to show connectivity. This confirms that the common pattern has not only correlated the patterns of the two ROIs on every time point, but it has also added temporal correlations to the patterns of the two ROIs making them fluctuate similarly over time (which is key for our model-based connectivity). We also observed that this spurious connectivity for 1-model RCA was not specific to the particular model we used and remained when using any arbitrarily defined random models (results not shown). Specifically, we observed that even models unrelated to the representational structure of one of the two ROIs (e.g., position and/or semantic categories) could lead to false connectivity in 1-model RCA. This can be explained by the fact that the time-locked common input will make the RDMs of the two ROIs be more similar to each other and also to any random RDM.

Finally, despite the correlations imposed on the contents of representations and the temporal patterns across ROIs, the 2-model RCA (correctly) showed negative correlations between the ROIs after the common input suggesting no connectivity (Figure 4A). This negative correlation can be explained by the fact that the two correlated representations will have a negative correlation when evaluated against two negatively correlated models. 2-model RCA is determined by the relationship between the neural RDMs of the two ROIs after projecting them on their relevant model RDMs. More specifically, it suggests that if the model RDMs for two ROIs do not correlate, or correlate negatively (suggesting distinct codes represented in each of them), they can remain immune to the added common noise. To test this hypothesis, we generated random models for the two ROIs of each simulated subject and calculated the level of increase in correlation/connectivity from before to after adding the common noise. Results showed a direct relationship between the similarities of the two models (and the corresponding ROIs) and the change in (2-model RCA) connectivity under the influence of the added noise (Figure 4B). Specifically, the more correlated the two models were, the larger the influence of adding correlated patterns. For negatively correlated models, like those in our simulation, adding correlated noise reduced the connectivity, so would not lead to spurious results. Therefore, 2-model RCA can be robust to shared noise or other common signals of no interest, but only if the 2-models are orthogonal or negatively correlated (and negative correlations are not interpreted).

#### Potential Solution

Both model-free and model-based RCA are affected by common-inputs to the two ROIs. This is particularly important for model-free RCA and 1-model RCA where it will always be the case, and should be taken into consideration when interpreting results. However, in 2-model RCA, where the two ROIs originally represented two distinct aspects of the task, the results were robust to the added common noise. This result was dependent on the chosen model RDMs not being positively correlated. Therefore, for cases where two regions are hypothesized to represent distinct information, the use of 2-model RCA with orthogonal or negatively correlated models can avoid spurious connectivity caused by common patterns of activation such as correlated noise.

### Simulation 3: Model-Based Representational Connectivity Analysis With Region of Interest-Specific Models Can Detect Transformation of Information Across Region of Interests

#### Problem Statement

There can be situations where the structure of the information is transformed from one ROI to the next. In fact, it seems unlikely that information remains intact (‘‘copied’’) between any two ROIs in the brain. Therefore, direct comparison of neural representations, as implemented in model-free RCA, can miss such potential connectivity simply because the statistical relationship may be lost in transformation. However, model-based RCA may allow us to detect the connectivity between two areas, which encode distinct information, based on their temporal statistical congruency. Below we simulate two ROIs that represent two distinct aspects of information, with dynamics that are either temporally congruent or incongruent between ROIs. Specifically, the information about the stimulus position initially appears in the source ROI (V2) and is followed by the semantic-category information which appears in the destination ROI (ITC)^2^. This scenario resembles a study which found evidence in support of causal information transfer and transformation from early visual to ITC areas (Clarke et al., 2018). As the detection of connectivity using model-based RCA with ROI-specific models also needs the adoption of correct model for each ROI, because the information is transformed, we also examine the effect of choosing the correct model for each ROI.

#### Simulation Details

**FIGURE 5:**
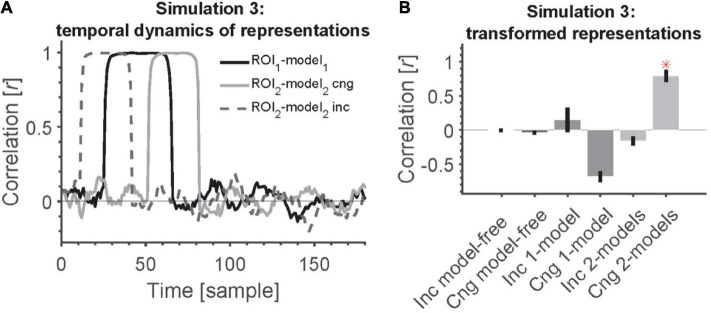
2-model RCA allows us to detect transformation of information if the temporal dynamics across ROIs are statistically related/congruent. **(A)** Time course of information encoding in the two ROIs at a delay of 20 samples [congruent (cng), solid gray line] and a delay of –20 from ROI 1 to ROI 2 [incongruent (inc), dashed gray line]. The delay was variable across simulated subjects. The time courses show the correlation between each ROI and its corresponding model (position model for ROI 1 and semantic-category model for ROI 2). **(B)** Transformation of information across ROIs causes all model-free and model-based RCAs to miss the connectivity except when the information appears congruently across ROIs (first in ROI 1 followed by ROI 2) and using 2-model RCA. Red asterisk shows significant above-chance correlation value (connectivity) as evaluated by one-sided Wilcoxon’s signed rank test against zero.

We used simulations to investigate the transformation of information using model-based RCA. The details of information representation in the two ROIs in this simulation are identical to Simulation 2, with the exception that the information does not appear throughout the simulation window but rather for a fixed period of time in each ROI (samples 30–60 in ROI 1; solid black curve in Figure 5A). There was a delay of ±20 samples between ROIs 1 and 2 (positive for congruent and negative for incongruent case) which was jittered between 0 and 10 samples (uniform random distribution) across the simulated subjects (*N* = 20). This led to information appearing in ROI 1 before ROI 2 in congruent cases, and in ROI 2 before ROI 1 in incongruent cases (Figure 5A). Specifically, patterns could appear between samples of 40 and 80 in ROI 2 in the congruent case and between samples of 0 and 40 in ROI 2 in the incongruent case. The activity patterns of the two ROIs did not contain any information in the samples outside the mentioned windows. Note that similar to the previous simulations, the information which was dominantly represented in the two ROIs was different [position encoding in V2 (source) and semantic category encoding in ITC (destination)]. This scenario simulated information flow from the source to the destination area that has been evaluated in previous studies using both model-free and model-based RCA (Goddard et al., 2016, 2021; Clarke et al., 2018; Karimi-Rouzbahani, 2018; Karimi-Rouzbahani et al., 2019, 2021a). In this analysis, the onset of information in each ROI predicts the direction of information transfer (e.g., potential information flow from V2 to ITC). Here we only evaluate the feed-forward information flow/connectivity from ROI 1 to ROI 2 (e.g., as in the ventral visual stream) and not vice versa. In testing the connectivity for both model-free and model-based RCA methods, we set the analysis delay-time (i.e., lag) between ROIs to be 20 (no jitter) for all our subjects. This parameter is usually set by the researcher and fixed across subjects (Goddard et al., 2016, 2021; Karimi-Rouzbahani, 2018; Karimi-Rouzbahani et al., 2019, 2021a). We performed model-free RCA by calculating the direct correlation between the RDM of the source ROI at time *t* and the RDM of the destination ROI at time *t*+τ where τ refers to the delay (= 20 samples) and then averaged the time course of correlations within each subject. For model-based RCA, we calculated the correlation between the neural and model RDMs for each ROI on every time point as in Simulation 2 (note that we considered the two cases of having one model RDM or two different model RDMs), shifted the model-correlation time course of ROI 2 by 20 (jittered between 0 and 10 samples) relative to ROI 1, and computed their correlation coefficient. In both model-free and model-based analyses, the incorporation of the delay compensated for the inter-ROI delay in the data.

Our assumption here is that two ROIs that encode/represent statistically unrelated information can be considered connected if their temporal information-encoding profiles are statistically related/congruent (representations appear in the destination after the source ROI at around the hypothesized delay). We ask whether such a relationship would be detected using model-free, 1-model and 2-model RCA.

#### Simulation Results

Figure 5A shows the time courses of correlations between the RDM of each ROI with its corresponding specific model RDM. In congruent trials, correlations between RDMs from ROI1 and model1 (black solid curve) peak reliably earlier than correlations between ROI2 and model2 RDMs (gray solid curve).

Simulation results show that model-free RCA did not detect any connectivity between the two ROIs (Figure 5B). The 1-model RCA also failed to detect the connectivity whether the representations in ROIs appeared congruently or incongruently. The reason is that the representations that were transformed from the source to the destination ROI no longer matched the common model in the destination ROI (we used the model RDM from ROI 1 for both ROIs). The 2-model RCA also failed to detect the connectivity when the representations appeared incongruently across the ROIs (first in the destination followed by the source) because information time courses in one did not reliably follow the other according to the hypothesized lag. However, the 2-model RCA could detect the connectivity when the representations appeared congruently across the ROIs. Therefore, for the transformed information to be detected, one needs to have both accurate models of information representations as well as correct prior knowledge about temporal dynamics and direction of information flow across ROIs.

Note that in these simulations, we incorporated the delay in our analysis and the two ROIs followed the temporal profiles of representations shown in Figure 5A. Therefore, the absence of connectivity in the model-free and 1-model RCA cannot be explained by the fact that we used lagged correlations in the 2 model case. Specifically, we incorporated the delay in ***all*** our RCA measures here to avoid any systematic difference in RCA across methods.

#### Potential Solutions

Model-free RCA is only sensitive to direct statistical relationship between neural RDMs, and fails to detect the connectivity if the two ROIs do not statistically relate. However, 2-model RCA allows detection of congruent inter-ROI statistical dependencies by having models that capture the representational structure of each ROI. Importantly, as 2-model RCA relies on hypotheses about the representations in source and destination areas, it will be less affected by confounders such as noise which are generally represented similarly across the two ROIs. Similar to the observation made in Simulation 2, it might be that common task-irrelevant patterns in both ROIs obscure the shared information as captured by 1-model RCA or the transformation of information as captured by 2-model RCA. A solution to this would be to remove their contribution by regressing out the RDM of the common pattern from the RDM of each ROI at each time-point. However, for this one needs the knowledge about the structure of the common patterns, which is not often known *a priori*. Researchers should be aware of these limitations so that they can choose their analysis method and interpretation accordingly.

Another solution to the failure of model-free RCA to detect connectivity under transformed representations might be to use non-linear mapping functions. Such functions allow more flexible relationships to be detected between areas despite drastic transformation of the representational structure. Such non-linear mapping functions include distance correlation (Geerligs and Henson, 2016), projection to a Riemannian manifold (Shahbazi et al., 2021) or more general functions estimated by artificial neural networks (Anzellotti et al., 2017b). These potential solutions are not investigated here.

## Discussion and Conclusion

**FIGURE 6:**
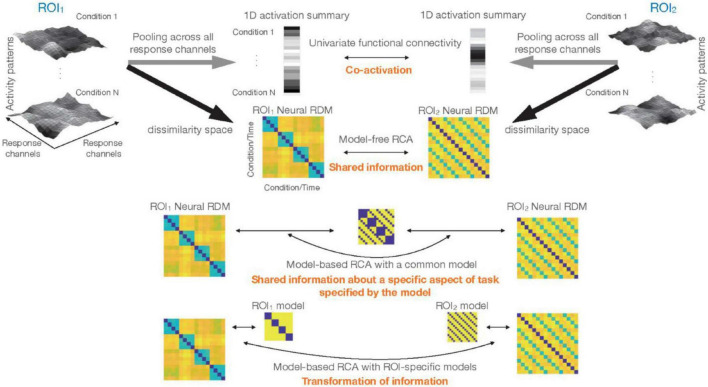
Different types of inference about functional connectivity: top left and top right show the response patterns for N experimental conditions in two ROIs. Larger activations in a voxel are shown by lighter colors. One classical approach would be to reduce the dimensionality of data in each ROI to 1, and summarize the rich patterns of activity by a single vector containing one number for each experimental condition (or time-point for the case of resting-state data). Significant correlation between these vectors implies co-activation, i.e., that activations in ROI1 and ROI2 co-vary. Multi-dimensional connectivity methods that we consider in this article characterize the response patterns for different conditions by a representational dissimilarity matrix (RDM). Direct comparison of the RDMs (model-free RCA) tests for shared information (i.e., whether the two sets of response patterns in the two ROIs have any shared information with regards to the experimental conditions). Incorporation of models, i.e., model-based RCA, when a common model is used for both ROIs (1-model RCA) tests for shared information about a specific aspect of task/stimuli. This hypothesis in RCA is specified in the ROI-common model. Finally, model-based RCA with ROI-specific models (2-model RCA) detects potential transformation of information.

Multi-dimensional connectivity is a rapidly developing area of brain connectivity analysis. One of the approaches to multi-dimensional connectivity is representational connectivity analysis (RCA). RCA quantifies the similarity of inter-relationship between the neural representations across experimental conditions for distributed patterns of activity of two brain regions (Kriegeskorte et al., 2008). This allows us to track “information” (by the representational geometry in the multi-dimensional response space) rather than the mere similarity of average response levels across two regions. Despite its versatility, a better understanding of the situations that can challenge and/or mislead RCA is needed. In this manuscript, we explain two main approaches of RCA. One is model-free RCA that directly compares the representational geometries of two brain ROIs. Model-free RCA can tell us whether two brain ROIs have any shared information in their multi-dimensional response patterns. The other is model-based RCA. In this article, we make a further distinction between two approaches to model-based RCA: using a single or multiple models. We think this distinction is important, since besides the difference in technical details and implementation, they entail different interpretations about regional interactions. The first variant of model-based RCA, which uses a common model (1-model RCA), tests whether the representational geometries of the two ROIs are similarly concordant to a hypothesized geometry (i.e., the model). This can tell us whether two brain regions have shared information with regard to a specific aspect of the stimuli/task. The other variant of model-based RCA uses ROI-specific models, which, with time-resolved data, tells us whether information in one region is transformed into different information in another region. Therefore, while this also pertains to functional connectivity, it does not explicitly get at shared information. Figure 6 provides an overview of the distinctions explained in the article.

Model-free and model-based RCA can potentially provide inconsistent results in certain circumstances. These inconsistencies depend on many factors, some of which are the spatiotemporal structure of neural representations and the choice of the model(s) used in the analysis, and inform interpretation. Here, we focused on three simulations where model-free and model-based RCA provided opposing connectivity results.

First, we simulated a situation where the neural representations across a pair of regions showed unrelated information. As expected, model-free RCA showed no connectivity between the pair of regions. Interestingly, however, we observed that using model-based RCA with an intermediate model, which contains information about the representations in both regions, can leave the false impression that the two regions are connected. Specifically, the two regions showed almost equal, positive and significant correlation to the intermediate model suggesting that from the “lens” of the selected model, the two regions appear to be connected.

There are a few considerations. First, although for simplicity we did not directly implement either of the two published methods of model-based RCA (Clarke et al., 2018; Karimi-Rouzbahani et al., 2021a), the problem we pointed out here can affect both those methods. This is because they compare the correlation between the models either explicitly (Clarke et al., 2018), or implicitly within the formulation of partial correlation (Karimi-Rouzbahani et al., 2021a). Second, although we used a two-component model for this simulation to simplify the interpretation, this situation is not limited to two-component models. In fact, any other models that share roughly equal amounts of variance with different components encoded in two areas would lead to a similar situation. Third, the false connectivity observed in this scenario is not driven by the specific similarity metric we used (i.e., Pearson’s linear correlation). Although different similarity metrics show different characteristics (Walther et al., 2016; Shahbazi et al., 2021), as long as the selected metric provides similar values for the similarity between two different neural and a given RDM model, the same effect will be observed. The reason is that all similarity metrics summarize a high-dimensional representational space into a single-dimensional space, which inevitably leads to loss of information. Finally, at the other end of the spectrum, there can be cases where two regions represent one or several very similar aspects of information, but they still look unrelated/disconnected through the lens of a particular model. However, this case seems less problematic since the main reason behind using model-based rather than model-free RCA is to limit the representations to the desired information (Karimi-Rouzbahani et al., 2021a). Nonetheless, it would be good practice to do perform both types of RCA (together with RSA information mapping) and to compare the results while being aware of the limitations and caveats of each.

In the second simulation, we modeled a situation where the addition of statistically related patterns of activity to a pair of statistically unrelated regions imposed a statistical relationship between them. This led to apparent connectivity in model-free RCA and when using 1-model RCA. However, the common pattern did not affect apparent connectivity when using 2-model RCA, as long as the two models were orthogonal and the two ROIs represented distinct information. Please note that the added common pattern can be non-structured or structured. Although we have seen that both common noise (non-structured) and structured patterns (data not shown) led to similar results, the structure of the common pattern can affect the connectivity as a result of interaction with the representations in the regions and models. It is also of note that the addition of common patterns does not always inflate the connectivity (e.g., in model-free or 1-model RCA); it can also decrease it leading to missing the connectivity. For example, if two regions are perfectly correlated, the addition of common noise (if not perfectly identical but only statistically related across regions) could lead to a decline in model-free RCA as a result of distorting the patterns. Generally, both model-free and model-based RCA can be affected by the noise as a result of the complex interaction between the representation in each region, the structure of the added pattern, the models, and the temporal dynamics of representations. Therefore, despite the situation shown in Simulation 2, these methods we are still far from remaining immune to common noise. We can, however, understand where we are most susceptible to it. One simple remedy for the effect of common patterns would be to regress out its contribution from the RDMs of the two ROIs prior to computing connectivity measures. This is in spirit similar to our recent implementation of model-based RCA (using partial correlation), where we partialled out the effect of additional low-level image statistics from the two regions under study (Karimi-Rouzbahani et al., 2021a). However, as the structure of the added pattern (noise or common structure of no interest) is usually unknown, this will not always be an option.

In the third simulation, we showed a situation where two regions encoded different types of information that were either temporally congruent or incongruent. In other words, the information initially appeared in one region and after some delay in the other region (temporally congruent). Model-based RCA with proper choices of models can capture this relationship. This may be useful as transformation of information seems an integral part of brain connectivity as it seems unlikely that information would remain intact from one brain region to another (Lahaye et al., 2003; Hlinka et al., 2011). Transformations of information have already been reported in visual system of human and monkey brain (DiCarlo et al., 2012; Kietzmann et al., 2019) and are implemented by other sensory hierarchies as well (Winkowski and Kanold, 2013). For example, it has been suggested that visual information is moved from low- to a high-dimensional space along the ventral visual stream and brought back to the low-dimensional space in later stages of the stream to compensate for variations of visual objects and form semantically categorized object clusters (DiCarlo et al., 2012; Karimi-Rouzbahani et al., 2017a,b). Using model-based RCA, previous work has found that information transforms from visual to semantic brain areas (Clarke et al., 2018). In our simulation, the drastic transformation of information simulated in Simulation 3 meant that the connectivity was missed by model-free RCA and 1-model RCA. However, 2-model model-based RCA detected the connectivity as a result of its simultaneous sensitivity to targeted region-specific information representation and the temporally congruent patterns of information representation. Therefore, a hypothesis-driven method of RCA allows us to detect information that is transformed as it passes between brain regions.

This simulation also demonstrated the importance of the delay in connectivity analysis matching the data. The delay in the analysis potentially captures the neural lag in information transfer in the brain (Cichy et al., 2014). The delay is generally set *a priori*, meaning that choice of improper delays (negative vs. positive; which also determines the direction of information) can lead to missing the connectivity. A more principled way of estimating the delay would be to partition the data and estimate the optimal delay from one half and apply it to the other half. However, this requires independent measurements of the same task in each subject. A more extended version of the RCA could be to perform Granger causality to examine Granger-causal relationships between areas as in previous studies (Goddard et al., 2016, 2021; Clarke et al., 2018; Karimi-Rouzbahani, 2018; Karimi-Rouzbahani et al., 2019; Kietzmann et al., 2019). That would also be subject to similar considerations. However, comparing the different approaches at a conceptual and mathematical level is beyond the scope of the current study.

It is generally desired that a connectivity method determines the transferred *content*, *direction*, and *temporal dynamics* of information flow. To that end, previous studies implemented techniques including partial correlation (Goddard et al., 2016, 2021; Karimi-Rouzbahani, 2018; Karimi-Rouzbahani et al., 2019, 2021a) and regression (Kietzmann et al., 2019), or tested for Granger causal relationship between areas (Goddard et al., 2016, 2021; Clarke et al., 2018; Karimi-Rouzbahani, 2018; Karimi-Rouzbahani et al., 2019), or used models to measure the contribution of one area to another in the direction of the task (Karimi-Rouzbahani et al., 2021a) or incorporated autoregressive approaches to estimate proper delay between areas (Clarke et al., 2018). In our most recent effort, to bring together the advantages of the mentioned methods, we proposed a variant of model-based RCA which provided information about the content of the transferred information, its direction and temporal dynamics simultaneously (Karimi-Rouzbahani et al., 2021a). This method showed distinct dynamics and direction of face familiarity-information flow across peri-frontal and peri-occipital cortices for different levels of perceptual uncertainty. Despite our minimalist approach in the current study, the insights and cautions provided by this work can be generalized to more complex implementations of RCA as well.

Additionally, one could also consider other extensions to model-free RCA. Similar to “information connectivity” (Coutanche, 2013) where multi-dimensional connectivity is established by correlating time series of classification-accuracies across regions, one can compare time courses of the exemplar discriminability index (EDI, Nili et al., 2020) across regions. EDI is a model-free RSA statistic in each region and quantifies the extent to which different experimental conditions elicit distinct patterns of activation. Similar to the implementation of model-free RCA, however, this definition of model-free RCA also does not shed light into the content of shared information.

One limitation of the current study is that we only evaluated connectivity using linear, rather than non-linear, relationships. While this simplification allowed us to make more intuitive predictions about the relationship between brain responses and the models, a more general approach would be to incorporate non-linear connectivity between areas as well. While we believe that the cases evaluated in Simulations 1 and 2 will not be affected by using a non-linear connectivity metric, non-linear mapping functions in Simulation 3 (Geerligs and Henson, 2016; Anzellotti et al., 2017b; Basti et al., 2020; Shahbazi et al., 2021) may allow for detecting non-linear relationships between areas. Therefore, future studies will need to evaluate the impact of non-linear mapping functions in RCA.

This work takes initial steps toward better characterization of the model-free and model-based RCA approaches that have been increasingly used in recent years. We tried to make the simulations as general and ideal as possible (no nuisance factors, e.g., measurement noise, leakage incorporated), so that the insights can be generalized to different implementations of the two general classes of model-free and model-based RCA. Therefore, the points made here can provide insight when studying brain connectivity using variety of neural recording modalities such as EEG, MEG, multi-electrode electrophysiology, and fMRI. Specifically, apart from Simulation 1, which presents a conceptual point applicable to all multivariate imaging/recording modalities, the methods implemented in Simulations 2 and 3 can directly be applied to EEG and MEG data.

## Data Availability Statement

The dataset used in this study is auto-generated using the Matlab script available at https://osf.io/3nxfa/ which generates the simulation figures as well.

## Author Contributions

HK-R: conceptualization, methodology, software, formal analysis, funding acquisition, writing – original draft, and writing – review and editing. AW and RH: conceptualization, methodology, and writing – review and editing. HN: conceptualization, methodology, software, formal analysis, writing – original draft, and writing – review and editing. All authors contributed to the article and approved the submitted version.

## Conflict of Interest

The authors declare that the research was conducted in the absence of any commercial or financial relationships that could be construed as a potential conflict of interest. The reviewer AC declared a shared affiliation, with several of the authors, HK-R, AW, and RH, to the handling editor at the time of the review.

## Publisher’s Note

All claims expressed in this article are solely those of the authors and do not necessarily represent those of their affiliated organizations, or those of the publisher, the editors and the reviewers. Any product that may be evaluated in this article, or claim that may be made by its manufacturer, is not guaranteed or endorsed by the publisher.

## Footnotes

1 We use the term “model” in a general sense: it can be a conceptual model, a computational model or a third brain region, etc.

2 We consider the case where the information is reliably transferred from one ROI to another as temporally congruent and cases where there is no transfer of information or the transformation is not reliable/consistent as temporally incongruent.
