# Erratum: Caveats and Nuances of Model-Based and Model-Free Representational Connectivity Analysis

**DOI:** 10.3389/fnins.2022.957057

**Published:** 2022-06-23

**Authors:** Frontiers Production Office

**Affiliations:** Frontiers Media SA, Lausanne, Switzerland

**Keywords:** representational connectivity analysis, multi-dimensional connectivity, functional connectivity, multivariate pattern analysis, representational similarity analysis

Due to a production error, there was a mistake in Figures 3, **5**, **6** in the published article. The correct Figures 3, **5**, **6** appear below.

**Figure 3:**
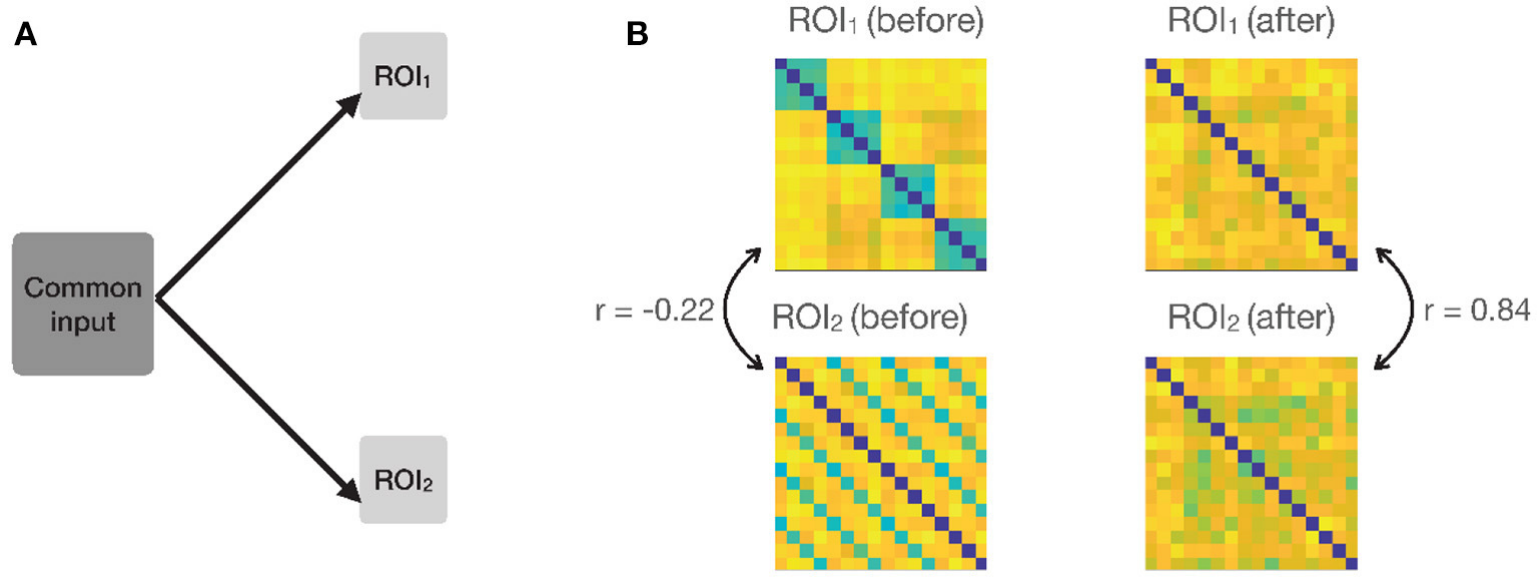
**(A)** Simulation settings, a common input is added to the responses in both ROIs, so that the patterns in the two ROIs covary at each time-point. **(B)** Example RDMs from the two ROIs before and after adding the common input. RDMs originally had a negative correlation (−0.22). The correlations went up from −0.22 to 0.84 due to the common input.

There was also a production error in the captions for Figures 3, 4. The caption for Figure 3 should have been applied to Figure 4, and the caption for Figure 4 should have been applied to Figure 3.

**Figure 4:**
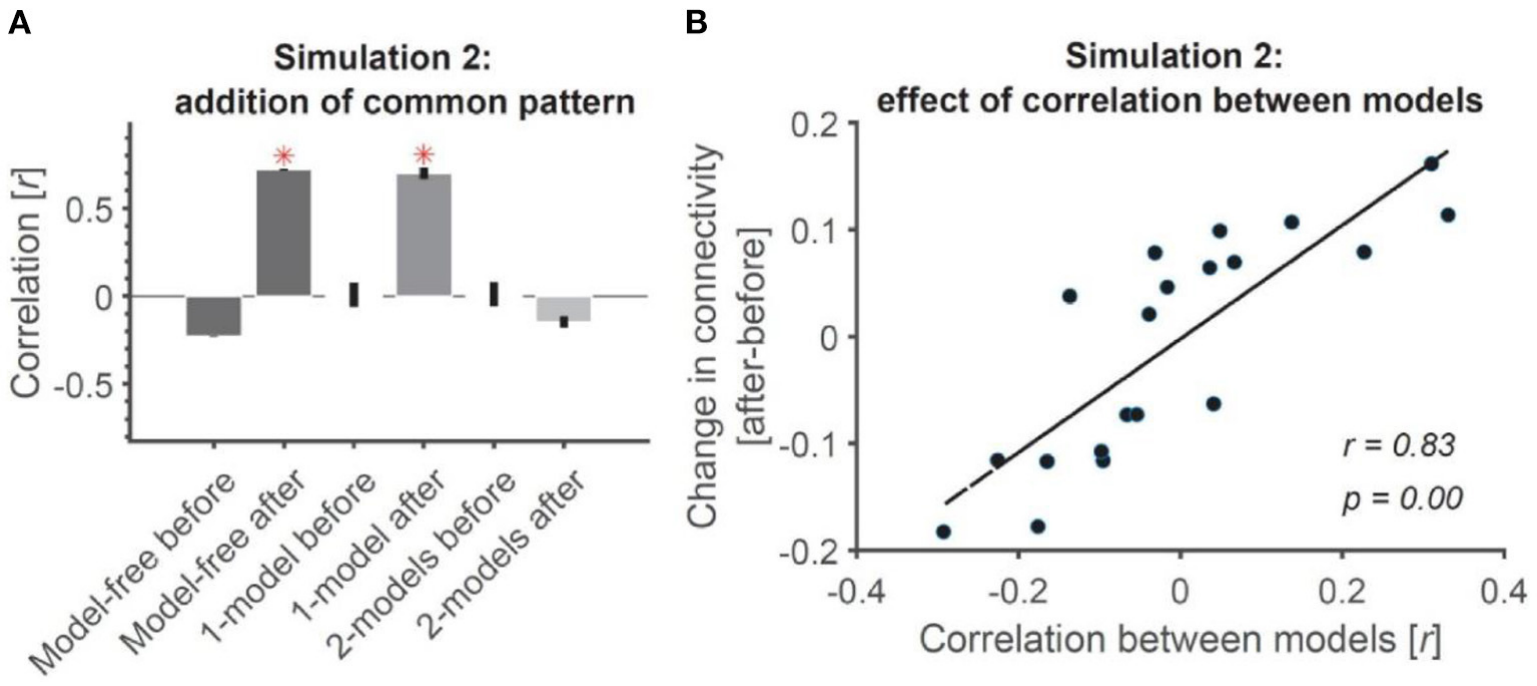
Addition of common pattern to pair of ROIs can make them look connected in model-free and 1-model RCA but not 2-model RCA if the ROIs are distinct enough. **(A)** Addition of common non-structured (noise) patterns to both ROIs leads to significant connectivity when evaluated using model-free and 1-model RCA, but not 2-model RCA because the two ROIs dominantly represent information that is negatively correlated (see **Figure 1B**). Red asterisks show significant above-chance correlation values (connectivity) as evaluated by one-sided Wilcoxon's signed rank test against zero. **(B)** The more distinct the dominant information represented across the ROIs, the less the effect of added common noise on their connectivity. Dots show the amount of change in connectivity as a function of correlation between the information represented in the two model RDMs used in 2-model RCA. Each dot represents data from a single simulated subject. The line shows the best linear fit to the data. The correlation and the significance of correlations are also shown as calculated using Pearson's linear correlation.

Additionally, there was an error in the section **“Simulation 3: Model-Based Representational Connectivity Analysis With Region of Interest-Specific Models Can Detect Transformation of Information Across Region of Interests**,” in the **“Simulation Results.”** The sentence “Simulation results show that model-free RCA did not detect any connectivity between the two ROIs (Figure 5A)” should read “Simulation results show that model-free RCA did not detect any connectivity between the two ROIs (Figure 5B).”

**Figure 5:**
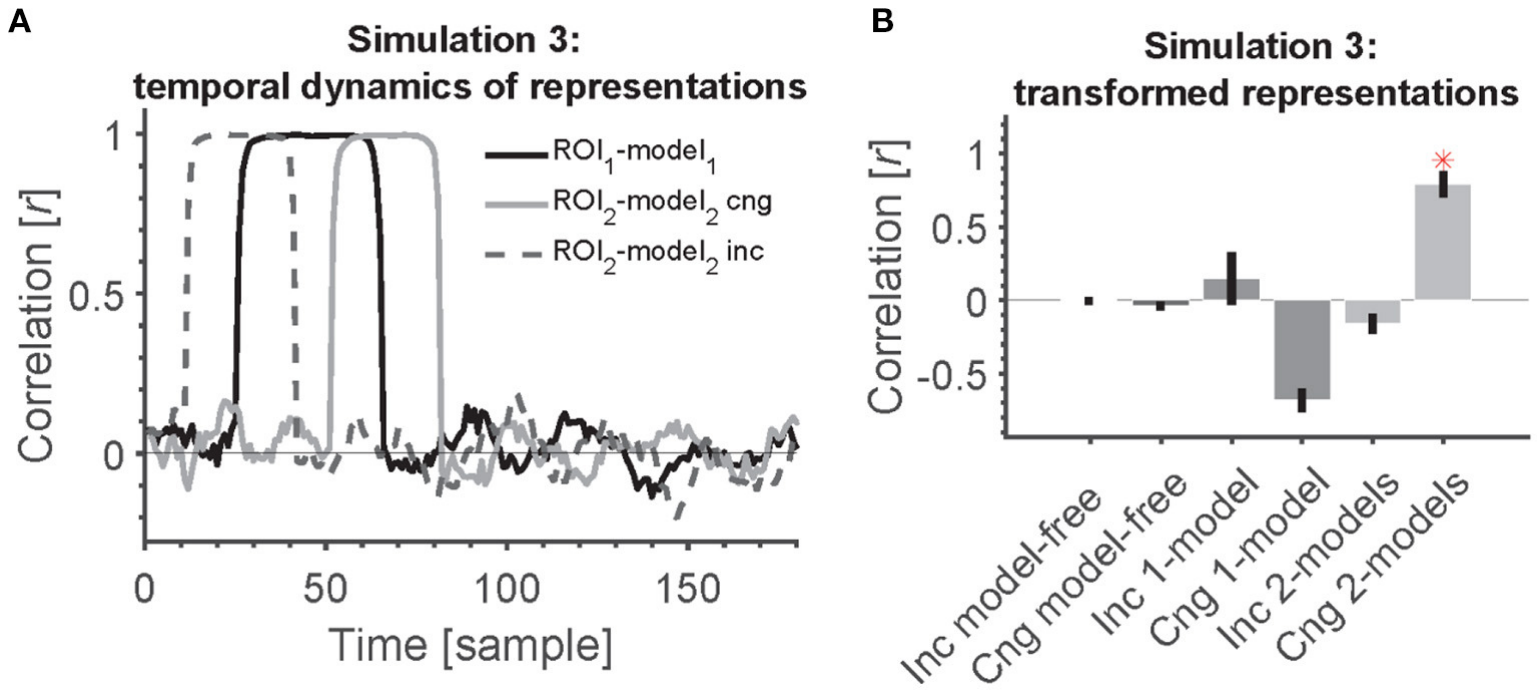
2-model RCA allows us to detect transformation of information if the temporal dynamics across ROIs are statistically related/congruent. **(A)** Time course of information encoding in the two ROIs at a delay of 20 samples [congruent (cng), solid gray line] and a delay of −20 from ROI 1 to ROI 2 [incongruent (inc), dashed gray line]. The delay was variable across simulated subjects. The time courses show the correlation between each ROI and its corresponding model (position model for ROI 1 and semantic-category model for ROI 2). **(B)** Transformation of information across ROIs causes all model-free and model-based RCAs to miss the connectivity except when the information appears congruently across ROIs (first in ROI 1 followed by ROI 2) and using 2-model RCA. Red asterisk shows significant above-chance correlation value (connectivity) as evaluated by one-sided Wilcoxon's signed rank test against zero.

**Figure 6:**
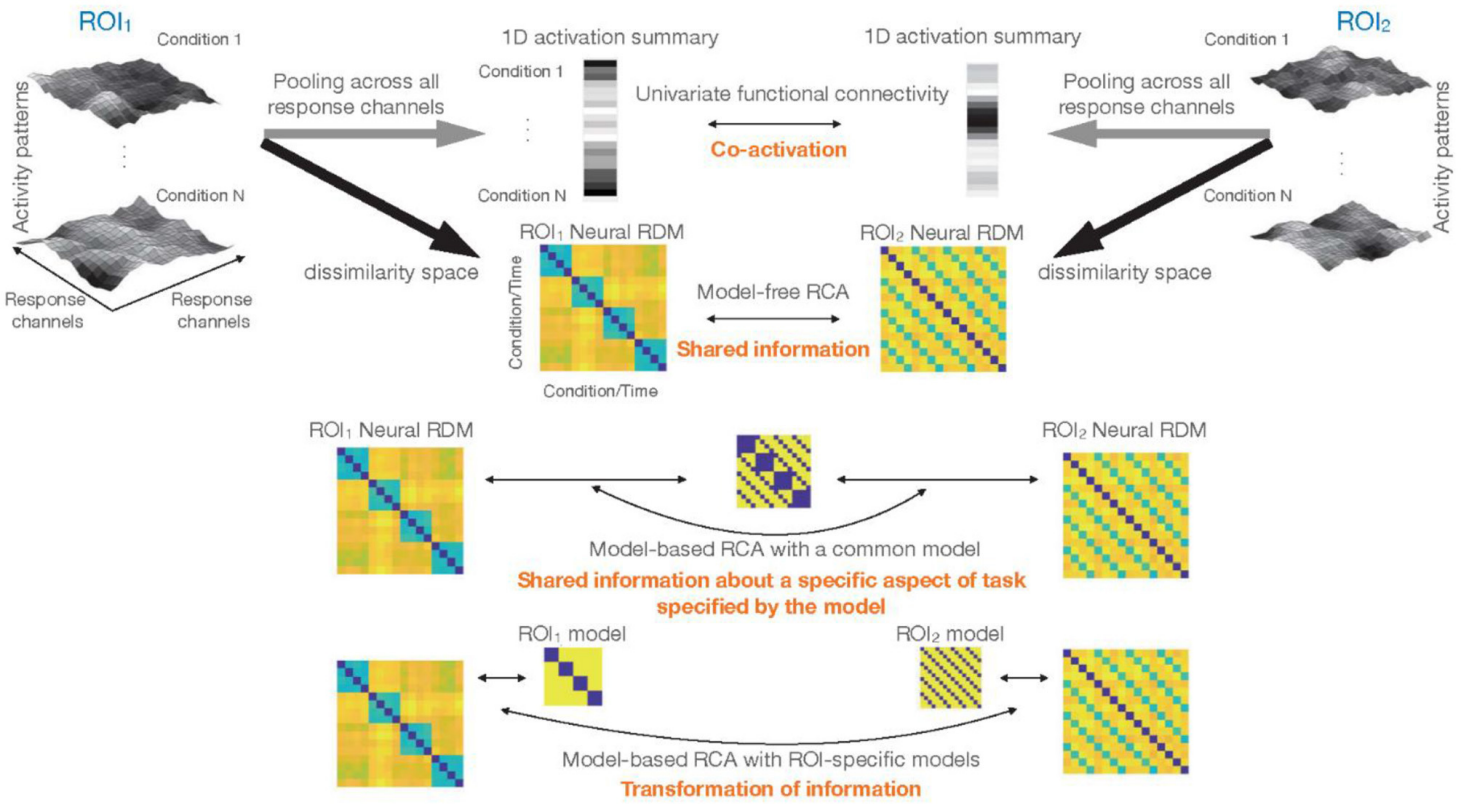
Different types of inference about functional connectivity: top left and top right show the response patterns for N experimental conditions in two ROIs. Larger activations in a voxel are shown by lighter colors. One classical approach would be to reduce the dimensionality of data in each ROI to 1, and summarize the rich patterns of activity by a single vector containing one number for each experimental condition (or time-point for the case of resting-state data). Significant correlation between these vectors implies co-activation, i.e., that activations in ROI1 and ROI2 co-vary. Multi-dimensional connectivity methods that we consider in this article characterize the response patterns for different conditions by a representational dissimilarity matrix (RDM). Direct comparison of the RDMs (model-free RCA) tests for shared information (i.e., whether the two sets of response patterns in the two ROIs have any shared information with regards to the experimental conditions). Incorporation of models, i.e., model-based RCA, when a common model is used for both ROIs (1-model RCA) tests for shared information about a specific aspect of task/stimuli. This hypothesis in RCA is specified in the ROI-common model. Finally, model-based RCA with ROI-specific models (2-model RCA) detects potential transformation of information.

The publisher apologizes for this mistake. The original article has been updated.

## Publisher's Note

All claims expressed in this article are solely those of the authors and do not necessarily represent those of their affiliated organizations, or those of the publisher, the editors and the reviewers. Any product that may be evaluated in this article, or claim that may be made by its manufacturer, is not guaranteed or endorsed by the publisher.

